# Epidemic Percolation Networks, Epidemic Outcomes, and Interventions

**DOI:** 10.1155/2011/543520

**Published:** 2011-02-21

**Authors:** Eben Kenah, Joel C. Miller

**Affiliations:** ^1^Department of Biostatistics, School of Public Health, University of Washington, Seattle, WA 98195-7232, USA; ^2^Center for Communicable Disease Dynamics, Department of Epidemiology, Harvard School of Public Health, Boston, MA 02115, USA; ^3^Fogarty International Center, National Institutes of Health, Bethesda, MD 20892, USA

## Abstract

Epidemic percolation networks (EPNs) are directed random networks that can be used to analyze stochastic “Susceptible-Infectious-Removed” (SIR) and “Susceptible-Exposed-Infectious-Removed” (SEIR) epidemic models, unifying and generalizing previous uses of networks and branching processes to analyze mass-action and network-based S(E)IR models. This paper explains the fundamental concepts underlying the definition and use of EPNs, using them to build intuition about the final outcomes of epidemics. We then show how EPNs provide a novel and useful perspective on the design of vaccination strategies.

## 1. Introduction

With the continual improvement of computing power, individual-based models of infectious disease spread have become more popular. These models allow us to incorporate stochastic effects, and individual-scale detail in ways that cannot be captured in more traditional models. In this paper, we review a framework based on directed random networks that unifies a range of individual-based models in closed populations, simplifying their analysis. We then show how this framework provides a new and potentially useful perspective on the design of vaccination strategies.

Directed random networks that we call *epidemic percolation networks* (EPNs) can be used to understand the final outcomes of stochastic “Susceptible-Infected-Removed” (SIR) and “Susceptible-Exposed-Infectious-Removed” (SEIR) epidemic models. In these models, susceptible persons are infected upon infectious contact with an infectious person. Once infected, they either become infectious immediately (in SIR models) or become infectious after a *latent period* (in SEIR models). Infectious persons eventually recover, after which they can neither infect others nor be infected. The number of persons infected in an epidemic is called the *size* of the epidemic, and the proportion of the population infected is called the *attack rate*. The average number of persons infected by a typical infectious person in the early stages of an epidemic is called the *basic reproductive number* and denoted by *R*_0_.

For simplicity, we assume the entire population is susceptible to infection at the beginning of an epidemic. When one or more persons are infected, there are two possible outcomes. In a *minor epidemic* or *outbreak*, the attack rate is negligible and transmission ceases because infected persons fail to make any infectious contacts. A *major epidemic* has a higher attack rate and transmission ceases because infected persons make infectious contact with previously infected persons. When *R*_0_ ≤ 1, major epidemics have probability zero. When *R*_0_ > 1, both major and minor epidemics can occur, and the probability and attack rate of a major epidemic both increase as *R*_0_ increases. (Mathematically, the distinction between major and minor epidemics is exact only in the limit of an infinite population size. In a finite population, there is a bimodal distribution of epidemic sizes when *R*_0_ > 1.) This pattern was first recognized in the 1950s [1], and it has proven to hold for almost all stochastic S(E)IR epidemic models, including mass-action and network-based models. 

The idea of using networks to represent the final outcomes of stochastic epidemic models developed separately for *mass-action* models and *network-based* models. In a mass-action model, an infectious person *i* can make infectious contact with any other person, but the probability of infectious contact from *i* to any given *j* ≠ *i* is inversely proportional to the population size. In a network-based model, infection is transmitted across the edges of a *contact network*. An infectious person *i* can make infectious contact with *j* ≠ *i* only if there is an edge connecting *i* and *j* in the contact network. The number of neighbors of *i* and the probabilities of infectious contact from *i* to his or her neighbors when *i* is infectious are independent of the population size. Both mass-action and network-based models can be analyzed using EPNs. Related concepts have been used previously [2–7], generally as a theoretical tool for proving rigorous results. In fact, this approach deserves more attention as a general framework for exploring S(E)IR epidemics: it provides a powerful theoretical tool, efficient numerical methods, and a guide to vaccination strategy design. In this paper, we review the fundamental concepts underlying the definition and use of EPNs and give examples of their use in designing efficient vaccination strategies for both mass-action and network-based epidemic models.

## 2. Epidemic Percolation Networks

 Informally, a single realization of the EPN is generated by considering each individual *i* separately, imagining *i* is infected, and drawing an arrow from *i* to all persons with whom he or she makes infectious contact. This gives us four possible edges in each unordered pair of nodes *i* and *j*:

1. no edge between *i* and *j*,
2. a directed edge from *i* to *j* (*i* → *j*),
3. a directed edge from *j* to *i* (*i* ← *j*),
4. an undirected edge between *i* and *j* (*i*↔*j*).

An outgoing or undirected edge from *i* to *j* indicates that *i* will make infectious contact with *j* if *i* is ever infected. If *i* is infected and *j* is at the end of one of the outgoing or undirected edges from *i*, then *j* is either infected by *i* or infected from another source prior to receiving infectious contact from *i*. In either case, *j* is infected before *i* recovers from infection. If person *j* is infected, then all persons at the end of an outgoing or undirected edge starting from *j* will be infected before *j* recovers from infection, and so on. Eventually, all persons connected to *i* by a series of outgoing or undirected edges will be infected.

In the rest of this section, we formally define a general stochastic SEIR model, define its EPN, and describe the epidemic threshold in terms of the emergence of giant components in the EPN.

### 2.1. General Stochastic SEIR Model

 Consider a closed population of *n* individuals assigned indices 1,…, *n*. Each individual is in one of four possible states: susceptible (S), exposed (E), infectious (I), or removed (R). Person *i* moves from S to E at his or her *infection time t*_*i*_, with *t*_*i*_ = *∞* if *i* is never infected. After infection, *i* has a *latent period* of length *ɛ*_*i*_, during which he or she is infected but not infectious. At time *t*_*i*_ + *ɛ*_*i*_, *i* moves from E to I, beginning an *infectious period* of length *ι*_*i*_. At time *t*_*i*_ + *r*_*i*_, where *r*_*i*_ = *ɛ*_*i*_ + *ι*_*i*_ is the *recovery period*, *i* moves from I to R. Once in R, *i* can no longer infect others or be infected. The latent period is a nonnegative random variable with cumulative distribution function (cdf) *F*_*i*_^E^(*ɛ*) and the infectious period is a strictly positive random variable with cdf *F*_*i*_^I^(*ι*). The recovery period is finite with probability one. An SIR model is an SEIR model where *ɛ*_*i*_ = 0 with probability one for all *i*.

An epidemic begins with one or more persons infected from outside the population, which we call *initial infections*. After becoming infectious at time *t*_*i*_ + *ɛ*_*i*_, person *i* makes infectious contact with *j* ≠ *i* at time *t*_*ij*_ = *t*_*i*_ + *ɛ*_*i*_ + *τ*_ij_*, where the *infectious contact interval τ*_ij_* is a strictly positive random variable with *τ*_ij_* = *∞* if infectious contact never occurs. Since infectious contact must occur while *i* is infectious or never, *τ*_ij_* ∈ (0, *ι*_*i*_] or *τ*_ij_* = *∞*. We define infectious contact to be sufficient to cause infection in a susceptible person, so the infection time *t*_*j*_ ≤ *t*_*ij*_. Let *F*_ij_*(*τ* | *ι*_*i*_) denote the conditional cdf of *τ*_ij_* given *ι*_*i*_. *F*_ij_*(*τ* | *ι*_*i*_) may depend on properties of *i* and *j* (such as age, immune status, contact intensity, etc.).

The general stochastic SEIR model can be turned into almost any standard epidemic model by choosing appropriate *F*_*i*_^E^(*ɛ*), *F*_*i*_^I^(*ι*), and *F*_ij_*(*τ* | *ι*_*i*_).

**Example 1**

In the stochastic Kermack-McKendrick SIR model for a population of size *n*, infectious persons have a constant hazard *μ* of recovery and there is a constant hazard *β*(*n*−1)^−1^ of infectious contact in every infectious-susceptible pair. This model can be obtained by taking

(1) $$\begin{array}{c} F_{i}^{\text{I}}\left(\iota \right)=1-e^{-\mu \iota },\\ F_{ij}^{*}\left(\tau \;|\;\iota_{i}\right)=\{\begin{array}{cc} 1-e^{-\beta \tau /\left(n-1\right)} & \text{if}\;\;\tau \in \left(0,\iota_{i}\right],\\ 1-e^{-\beta \iota_{i}/\left(n-1\right)} & \text{if}\;\;\tau \in \left(\iota_{i},\infty \right). \end{array} \end{array}$$

**Example 2**

In the network-based analogue of the Kermack-McKendrick model, infection is transmitted across the edges of a contact network. It has the same infectious period distribution as the mass-action model but a constant hazard *β* of infectious contact that does not depend on *n*. Thus,

(2) $$F_{ij}^{*}\left(\tau \;|\;\iota_{i}\right)=\{\begin{array}{cc} 1-e^{-\beta \tau } & \text{if}\;\;\tau \in \left(0,\iota_{i}\right],\\ 1-e^{-\beta \iota_{i}} & \text{if}\;\;\tau \in \left(\iota_{i},\infty \right), \end{array}$$

whenever *i* and *j* are connected in the contact network. When *i* and *j* are not connected, *τ*_ij_* = *∞* with probability one.

### 2.2. Time Homogeneity and the EPN

 The general stochastic epidemic model is *time-homogeneous*, which means that the latent period, infectious period, and infectious contact interval distributions are specified *a priori*. This gives us two equivalent ways to run the model.

1. First, we can sample “on the fly” for each new infection *i* by generating a latent period *ɛ*_*i*_ and an infectious period *ι*_*i*_ and then sampling *τ*_ij_* from its conditional distribution given *ι*_*i*_ for each *j* ≠ *i*.
2. Second, we can sample *a priori* by generating *ɛ*_*i*_ and *ι*_*i*_ for each *i* and then sampling *τ*_ij_* from the appropriate conditional distribution for each ordered pair *ij* before starting the epidemic. We then look up these values as we need them to run the model.

Sampling on the fly is more efficient if the goal is to produce just a single epidemic realization, but sampling *a priori* provides information about many possible epidemics and leads to the definition of the EPN. A single realization of the EPN can be generated as follows.

(1) For each individual *i*,
  - sample a latent period *ɛ*_*i*_ from *F*_*i*_^E^(*ɛ*),
  - sample an infectious period *ι*_*i*_ from *F*_*i*_^I^(*ι*),
  - for each *j* ≠ *i*, sample an infectious contact interval *τ*_ij_* from *F*_ij_*(*τ* | *ι*_*i*_).
(2) For each pair of individuals *i* and *j*,
  - if *τ*_ij_* < *∞* and *τ*_ji_* < *∞*, draw an undirected edge between *i* and *j*,
  - if *τ*_ij_* < *∞* and *τ*_ji_* = *∞*, draw a directed edge from *i* to *j*,
  - if *τ*_ij_* = *∞* and *τ*_ji_* < *∞*, draw a directed edge from *j* to *i*,
  - if *τ*_ij_* = *∞* and *τ*_ji_* = *∞*, draw no edge between *i* and *j*.

The time homogeneity assumption guarantees that *ɛ*_*i*_, *ι*_*i*_, and each *τ*_ij_* are chosen from the correct distributions regardless of when node *i* actually becomes infected. For a population of size *n*, there are up to 2^*n*(*n*+1)^ possible realizations of the EPN, each with a different edge set. The probability of each possible edge set is determined by the underlying SEIR model.

### 2.3. Degrees, Components, and Epidemics

 The most important properties of the EPN are its degree distribution and its component size distributions. The *indegree*, *outdegree*, and *undirected degree* of node *i* are the number of incoming, outgoing, and undirected edges incident to *i*. The degree distribution of the EPN is the joint distribution of these degrees over the nodes in the network. The *in-component* of node *i* is the set of nodes from which *i* can be reached by following a series of edges in the correct direction. The *out-component* of node *i* is the set of nodes that can be reached from *i* by following a series of edges in the correct direction. In both definitions, undirected edges can be crossed in either direction and the in- and out-components of node *i* include *i* itself. If any node in the in-component of *i* is infected, then *i* will be infected eventually. If *i* is infected, then every node in the out-component of *i* will be infected eventually. Since the EPN is a random network, each node does not have fixed in-, out-, or undirected degrees or fixed in- and out-components (though these are fixed in any single realization of the EPN). However, the distribution of the out-component sizes of node *i* is exactly the same as the distribution of epidemic sizes obtained in repeated runs of the corresponding S(E)IR model with *i* as the initial infection [8, 9].

**Figure 1:**
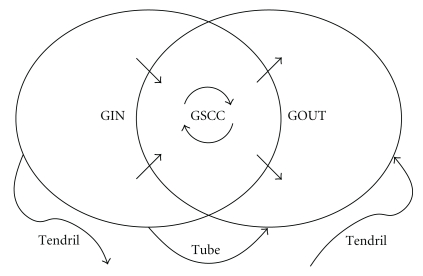
Schematic diagram of the giant components of an EPN. Note that the GIN and GOUT both include the GSCC. Tendrils are directed paths out of the GIN or into the GOUT that do not enter or leave the GSCC; a tube is a tendril that goes from the GIN to the GOUT. An initial infection in the GIN will lead to the infection of the entire GOUT (including the GSCC). If the initial infection is outside the GSCC, it will also infect a few tendrils or tubes and a few nodes in the GIN outside the GSCC. Since these are small components, their existence does not affect the calculation of the asymptotic probability and attack rate of a major epidemic. Adapted from [19].

This property of the EPN has several useful consequences. The epidemic threshold of an S(E)IR model corresponds to the emergence of *giant components* in the EPN. A *strongly connected component* is a group of nodes in which each node can be reached from every other node by following a series of edges. The nodes in a strongly connected component all have the same in-component and the same out-component. The EPN for a model below the epidemic threshold consists of many small strongly connected components. The EPN for a model above the epidemic threshold consists of a single *giant strongly connected component* (GSCC) and many small strongly connected components. The in- and out-components of nodes in the GSCC are called the *giant in-component* (GIN) and the *giant out-component* (GOUT). A schematic diagram of the giant components is in Figure 1. If all initial infections occur outside the GIN, a minor epidemic occurs because the out-components of all initial infections are small. If an initial infection occurs in the GIN, infection spreads to the GSCC and to the rest of the GOUT, so there is a major epidemic. If a single initial infection is chosen randomly, the distribution of outbreak sizes is equal to the distribution of small out-component sizes and the probability of a major epidemic is equal to the proportion of the network contained in the GIN. No matter where a major epidemic starts, its attack rate is equal to the proportion of the network contained in the GOUT.

## 3. Analysis of Stochastic SEIR Models

 In the limit of large *n*, almost all realizations of the EPN have the same degree distribution and the same distribution of in-, out-, and strongly connected component sizes, so a great deal of information is contained in a single realization of the EPN for an S(E)IR model with a large population. For many models, the asymptotic distribution of small component sizes and the proportion of the network contained in each of the giant components—and hence the outbreak size distribution and the asymptotic probability and attack rate of a major epidemic—can be calculated using probability generating functions [8–10]. In this section, we show how the analysis of mass-action and network-based SEIR models using EPNs and probability generating functions is a generalization of earler methods. We also show that EPNs are a useful theoretical tool and a powerful numerical tool for simulating S(E)IR epidemics in closed populations.

To demonstrate the accuracy of the EPN framework, we compare theoretical predictions of the probability and attack rate of a major epidemic based on EPNs with observations from a series of simulations of mass-action and network-based models. These simulations were implemented in Python (http://www.python.org/) using the SciPy (http://www.scipy.org/) [11] and NetworkX (http://networkx.lanl.gov/) [12] packages. The code is available as online supplementary material (see Supplementary Material available online at doi:10.1155/2011/543520). 

### 3.1. EPNs for Mass-Action Models

 In mass-action SEIR models like Example 1, infectious contact is possible between any two individuals, but the probability of infectious contact is inversely proportional to the population size. (Mathematically, the cumulative hazard of infectious contact is inversely proportional to the population size, but the cumulative hazard and the probability are approximately equal for very small probabilities.) There is a long tradition of approximating the initial spread of infection using a branching process [1, 13–15]. In a branching process, one or more initial nodes have offspring, where the number of offspring is a random sample from a given discrete distribution. Their offspring have offspring according to the same distribution, and so on. The *total size* of the branching process is the total number of individuals produced. When the mean number of offspring is greater than one, there is a positive probability that the branching process “explodes,” continuing forever and producing an infinite population. The total size distribution and the explosion probability can be calculated using probability generating functions.

For mass-action models with independent infectiousness and susceptibility, the outbreak size distribution and the probability and attack rate of a major epidemic can be predicted using branching process approximations that become exact in the limit of large *n* [14]. In the “forward” branching process, the offspring of each infection are the people he or she infects. In the “backward” branching process, the offspring of each infection are the people who would have infected the parent had they been infected. Asymptotically, the outbreak size distribution is equal to the distribution of finite total sizes in the forward branching process, and the probability of an epidemic is equal to the probability that the forward branching process explodes. The attack rate of a major epidemic is asymptotically equal to the probability that the backward branching process explodes. For these models, the out-component size distribution in the EPN is identical to the total size distribution of the forward branching process and the in-component size distribution of the EPN is identical to the total size distribution of the backward branching process. Thus, the EPN predicts the same outbreak size distribution and probability and attack rate of a major epidemic as the branching process approximations. When infectiousness and susceptibility are not independent, the branching process approximations break down but the EPN predictions remain correct [9]. In this case, the probability generating functions in the EPN approach are similar to those of a branching process, but they allow the number of offspring in the first generation (i.e., the initial infections) to have a different distribution than the number of offspring in all subsequent generations.

**Figure 2:**
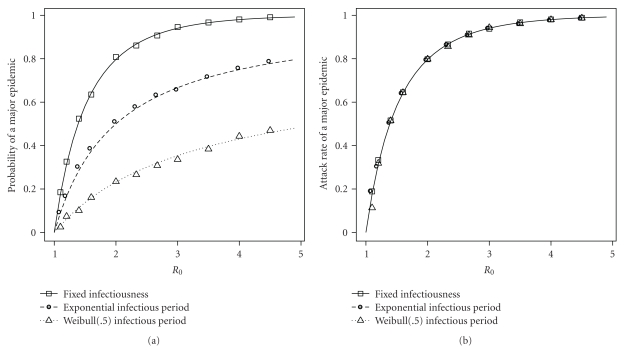
Major epidemic probabilities and attack rates in the mass-action models from Section 3.1. Lines represent theoretical predictions based on EPNs, and symbols represent observed probabilities and attack rates in simulations. The observed probability of a major epidemic is based on 1,000 runs of each model, where a major epidemic was defined as an epidemic with a final size ≥250. The observed attack rate is based on a single major epidemic. All models have a population of size 50,000. The predicted probability and attack rate of a major epidemic are equal only when the infectious period is fixed.

Figure 2 shows the observed and predicted probabilities and attack rates of a major epidemic in a series of mass-action SIR models. Each model had a population of 50,000. The observed probability of a major epidemic was the number of epidemics with a final size ≥250 out of 1,000 runs. The observed final size of an epidemic was based on a single major epidemic, which was defined as an epidemic with a final size ≥250. All models have a mean infectious period of one. There are three series of models: one with a fixed infectious period, one with an exponentially distributed infectious period, and one where the infectious period has a Weibull distribution with shape parameter 0.5. At each *R*_0_, these distributions produce different probabilities of a major epidemic but the same attack rate. The predicted probability and attack rate of a major epidemic are equal only when the infectious period is fixed.

### 3.2. EPNs for Network-Based Models

In a network-based SEIR model, infection is transmitted across the edges of a contact network. For network-based models, analysis via EPNs can be seen as a generalization of analysis via bond percolation models, first used to calculate the attack rate of a major epidemic [16] and later extended to the size distribution of minor epidemics and the probability of a major epidemic [17]. In this approach, each edge in the contact network is erased with probability 1 − *T*, where *T* is the marginal probability of infectious contact from an infected node to a neighbor. When infectiousness and susceptibility are constant, the distribution of minor epidemic sizes is equal to the distribution of small component sizes, the epidemic threshold corresponds to the emergence of a giant component in the posterasure contact network, and the probability and attack rate of a major epidemic are both equal to the proportion of the network contained in the giant component.

To illustrate this approach and its limitations, we generalize the network-based Kermack-McKendrick model from Example 2 by allowing it to have an arbitrary infectious period distribution. In the corresponding bond percolation model, each edge in the contact network would be retained independently with probability

(3) $$T=\overset{\infty }{\underset{0}{\int }}1-e^{-\beta \iota }\text{d}F^{\text{I}}\left(\iota \right),$$

where 1 − exp  (−*βι*) is the conditional probability of infectious contact given an infectious period of *ι* and d*F* ^I^(*ι*) represents integration or summation over the infectious period distribution. When the infectious period is fixed, the bond percolation model and the EPN predict exactly the same distribution of outbreak sizes and the same probability and attack rate of a major epidemic (which are equal in this case). However, the bond percolation model does not predict the correct outbreak size distribution or probability of an epidemic if the infectious period is variable.

**Example 3**

Consider a network-based Kermack-McKendrick model that has an exponential infectious period with mean one. The probability that a single initial infection with 2 neighbors fails to transmit infection is

(4) $$\overset{\infty }{\underset{0}{\int }}e^{-(2\beta +1)\iota }\;d\iota =\frac{1}{2\beta +1},$$

which is the probability that the corresponding node has an out-component of size one in the EPN. In the bond percolation model, (3) gives us *T* = *β*(*β*+1)^−1^, so the probability that both edges incident to the initial infection get erased is

(5) $${\left(1-T\right)}^{2}=\frac{1}{\beta^{2}+2\beta +1}<\frac{1}{2\beta +1}.$$

Thus, the bond percolation model underestimates the probability of an outbreak of size one. The bond percolation model treats infection of the two neighbors as independent events, but they are positively correlated because both are affected by the infectious period of the initial node. This limitation of the bond percolation model also affects contact networks that include directed edges, as considered in [18]. To see this, replace the undirected edges in this example with any combination of outgoing and undirected edges.

In this class of models, the bond percolation model overestimates the probability of a major epidemic whenever there is a variable infectious period [8, 10]. To demonstrate this, we conducted a series of simulations on Erdős-Rényi networks with mean degree 5. Each model had a population of 50,000. The observed probability of a major epidemic was the number of epidemics with a final size ≥250 out of 1,000 runs. The observed final size of an epidemic was based on a single major epidemic, defined as an epidemic with a final size ≥250. One series of simulations had a fixed infectious period of one, the second series had an exponentially distributed infectious period with mean one, and the third series had infectious persons transmit to all or none of their contacts. The first and last models define the upper and lower bounds, respectively, of the epidemic probability for models with independent infectiousness and susceptibility [10].

**Figure 3:**
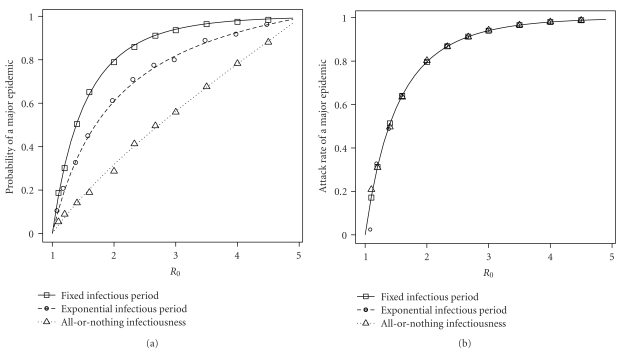
Major epidemic probabilities and attack rates in network-based models from Section 3.2. Lines represent theoretical predictions based on EPNs and symbols represent observed probabilities and attack rates in simulations. The observed probability of a major epidemic is based on 1,000 runs of each model, where a major epidemic was defined as having a final size ≥250. The observed attack rate is based on a single major epidemic. All models have a population of size 50,000 and an Erdős-Rényi contact network with mean degree 5. The predicted probability and attack rate of a major epidemic are equal only when the infectious period is fixed.

For a given *R*_0_, all three models have the same *T*, so they should have identical major epidemic probabilities and attack rates according to the bond percolation framework. In addition, the bond percolation framework implies that the probability and attack rate are always equal. However, Figure 3 clearly shows that the three models produce different major epidemic probabilities but equal attack rates. The probability and attack rate are equal only in models with a fixed infectious period. In models with variable infectiousness, the probabilities are lower than the attack rates. The EPN predictions of probability and attack rate are accurate for all of these models.

In these examples and the models considered in [17], there is variable infectiousness but constant susceptibility. When models have variable infectiousness and susceptibility, the bond percolation approach can predict the wrong attack rate for a major epidemic in addition to the wrong outbreak size distribution and probability of a major epidemic [10]. The EPN is very similar to the “locally dependent random graph” [4] for SIR epidemics on lattices, which was used to show that SIR models on lattices reduce to bond percolation processes if and only if the infectious period is constant [20]. In these and all other time-homogeneous S(E)IR models on networks, an analysis based on EPNs predicts the correct minor epidemic size distribution and the correct probability and attack rate of a major epidemic.

### 3.3. EPNs as a Theoretical Tool

 Most stochastic simulations of epidemic spread provide dynamic information about the spread of an epidemic. In contrast, a realization of an EPN is a static object, so many more mathematical tools are available to analyze it. To calculate the probability of a major epidemic, it suffices to calculate the proportion of nodes in the GIN. To calculate the attack rate, it suffices to calculate the proportion in the GOUT. In the infinite population limit, this is equivalent to calculating the probability that the EPN has an infinite path directed into or out of a randomly chosen node. This justifies the branching process approximation for mass-action models, and a similar approach is appropriate in networks without short cycles. These lead to analyses based on probability generating functions for the bond percolation model [17] and for EPNs [8–10].

More generally, however, we cannot use probability generating functions when the branches of the initial spread of infection intersect with asymptotically nonzero probability. Thus, we need different approaches to calculate the size and probability of an epidemic on a network with short cycles. This can be done numerically with EPNs as described below, but we can also use EPNs to make rigorous statements about the disease spread.

For example, if we want to analyze the impact a single individual has on an epidemic, a standard stochastic model would require many simulations. With an EPN approach, we are able to generate a realization of the EPN, including edges between all other nodes, and then consider the impact of each possible edge involving the targeted individual. This approach was used in [21] to investigate the impact of heterogeneity in the population. This paper compared two different random rules (with identical average) for assigning each node's infectiousness and susceptibility (independently of other nodes and each other), showing that the rule which provides a more homogeneous population results in larger and more probable epidemics. The approach was to choose a node *u* and consider any EPN realization created without edges involving *u*. Then the infectiousness and susceptibility of *u* would be assigned and the edges involving *u* chosen. It was then proven that regardless of how the remainder of the EPN was assigned, the size of in- and out-components would be maximized by the more homogeneous assignment rule.

### 3.4. EPNs as a Numerical Tool

 EPNs are a powerful numerical tool for the simulation and analysis of epidemics. Traditionally, the probability and attack rate of a major epidemic in an S(E)IR model are estimated by running the model repeatedly. For each run, we record whether a major epidemic occurred and, if so, we record the attack rate. Whether a major epidemic occurs is a binomial process where each run of the model is like a single coin flip. When an epidemic occurs, the size has only a small variation. Thus, repeated simulation produces an accurate estimate of the attack rate much faster than the probability. In a model with a sufficiently large population, the probability and attack rate of a major epidemic can be calculated with equal precision from a single realization of the EPN. Tarjan's algorithm [22] can be used to identify the GSCC, GIN, and GOUT in a time that is linear in the population size *n*. The sizes of the giant components vary by an amount of order ln  *n*, so the proportional error is small for large *n*.

**Figure 4:**
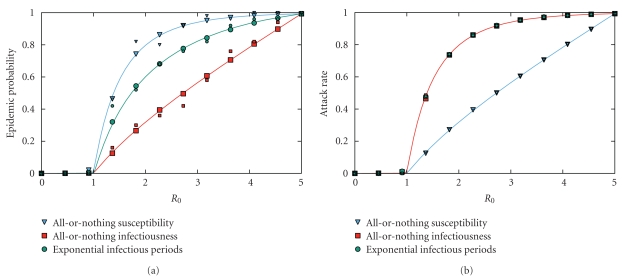
A comparison of the predictions from a single EPN with 50 simulations for three different epidemic processes on an Erdős-Rényi network of 50,000 nodes with average degree 5. The EPN results (large symbols) closely match the calculated predictions in the asymptotic limit. The simulated results (small symbols) compare well for size but poorly for probability. Generating an EPN is a much more efficient numerical method for estimating the probability of a major epidemic than simulation.

In Figure 4, we compare the result from a single EPN and the results from 50 simulations with the exact calculations in the infinite population limit. We consider three different models for the spread of a disease: exponentially distributed infectious periods, all-or-nothing infectiousness, and all-or-nothing susceptibility. In all-or-nothing infectiousness, a proportion of the population infects every susceptible neighbor, while the remainder infect none. In all-or-nothing susceptibility, a proportion become infected if any neighbor is ever infected, while the remainder are never infected. In the simulations, a single initial infection was chosen at random. At each *R*_0_, the calculations of a single EPN took approximately 1/3 the time of simulating 50 epidemics. We find that either approach does very well at predicting the attack rate. However, the probability of an epidemic is poorly predicted by the simulations because the convergence of a binomial process is relatively slow. If the population had short cycles, the exact calculations used to predict the probability and attack rate would fail. In that case, a single EPN would provide an accurate numerical prediction of the probability of a major epidemic much faster than repeated simulations.

Although our attention has focused on static quantities such as the probability or size of major epidemics, EPNs can also be used to calculate the dynamic spread of an epidemic. Returning to the generation algorithm described in Section 2.2, we can assign a latent period *ɛ*_*i*_ to each node *i*. For each ordered pair with *τ*_ij_* < *∞*, we can assign a time of *ɛ*_*i*_ + *τ*_ij_* to the edge from *i* to *j* in the EPN (similarly, if *τ*_ji_* < *∞*, assign a time of *ɛ*_*j*_ + *τ*_ji_* to the opposite direction). The time associated with each edge can be thought of as the “length” of the edge. If an epidemic begins with a single initial infection at time zero, the infection time of node *i* is simply the total length of the shortest path from the initial infection to node *i*. If no such path exists, *i* is never infected. These paths and their lengths can be found in time proportional to *n*ln  *n* using Dijkstra's algorithm [23]. Thus, EPNs provide an extremely efficient way to get complete runs of an epidemic model, including time dynamics as well as the final outcome. By choosing a different initial infection each time, many nearly independent runs of the SEIR model can be obtained from a single EPN.

## 4. Vaccination Strategies

 In this section, we show how EPNs provide a useful guide to the design of efficient vaccination strategies in mass-action and network-based SEIR models. For simplicity, we assume that we have a perfect vaccine that makes its recipients immune to infection. The effect of the vaccine can be represented by erasing all incoming, outgoing, and undirected edges incident to each vaccinated node in the EPN. Since a major epidemic is possible if and only if there is a GSCC, we hypothesized that vaccine should be targeted to nodes with a high probability of inclusion in the GSCC and a high number of connections to nodes in GSCC.

### 4.1. Vaccination in Mass-Action Models

 To test the effect of this targeting strategy in a mass-action model, we created a mass-action model with three subpopulations, A, B, and C, of equal size. Subpopulation A had high infectiousness but low susceptibility, subpopulation B had average infectiousness and susceptibility, and subpopulation C had low infectiousness but high susceptibility. Within subpopulation A, each node had a relative susceptibility that was exponentially distributed with mean one. Within subpopulation C, each node had a relative infectiousness that was exponentially distributed with mean one. Subpopulation A had the highest probability of being in the GIN, subpopulation B had the highest probability of being in the GSCC, and subpopulation C had the highest probability of being in the GOUT. With no vaccination, *R*_0_ = 2.14 and the probability and attack rate of a major epidemic are both equal to  .72. The EPN for this model is summarized in Table 1. Calculations of *R*_0_ and the probability and attack rate of a major epidemic at different vaccination fractions in each subpopulation were done using probability generating functions [9, 10].

**Results**

**Figure 5:**
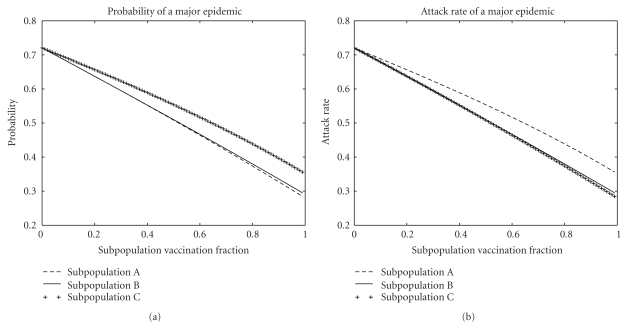
Effects of vaccination on the probability and attack rate of a major epidemic in the mass-action model from Section 4.1. Vaccinating subpopulation A, the most infectious and least susceptible, is optimal for reducing the probability. Vaccinating subpopulation C, the most susceptible and least infectious, is optimal for reducing the attack rate. Vaccinating subpopulation B, of average infectiousness and susceptibility, is nearly optimal for both.

**Figure 6:**
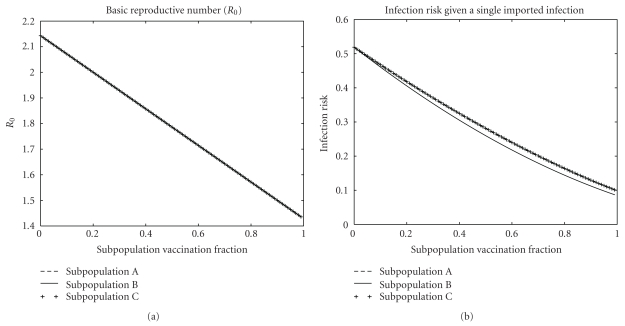
Effects of vaccination on *R*_0_ and the risk of infection given a single randomly chosen initial infection (the probability of a major epidemic times the attack rate) in the mass-action models from Section 4.1. Though all three vaccination strategies have the same effect on *R*_0_, vaccinating subpopulation B is optimal for reducing the overall risk of infection.

The results of the three vaccination strategies are shown in Figures 5 and 6. Vaccinating subpopulation A was optimal for reducing the probability of an epidemic because its members were the most infectious and the most likely to be in the GIN. Vaccinating subpopulation C was optimal for reducing the attack rate of an epidemic because its members were the most susceptible and the most likely to be in the GOUT. Vaccinating subpopulation B was nearly optimal for reducing both the probability and attack rate of a major epidemic because its members had the right combination of infectiousness and susceptibility to make them the most likely to be in the GSCC. Vaccinating any of the three subpopulations produced exactly the same effect on *R*_0_, but vaccinating subpopulation B was most effective in reducing the overall risk of infection given a single initial infection. The theory of EPNs gives us intuitive explanations for all of these effects.

### 4.2. Vaccination in Network-Based Models

 The standard approach to vaccination targeting in network-based models is to target nodes with high degree in the contact network [24, 25]. However, this approach ignores all information about the disease other than the contact network itself. An approach based on the EPN uses information about both the contact network and the epidemiological characteristics of the disease. In a series of epidemic models, we compared two vaccine-targeting strategies, each represented by a ranked list of nodes to vaccinate. For the first strategy, we ranked the nodes by degree in the contact network. For the second, we generated a single realization of the EPN and ranked nodes in the GSCC by the number of edges connecting them to other nodes in the GSCC, ignoring direction. Nodes outside the GSCC were placed in random order at the bottom of the list. For a vaccination fraction of *v*, the first *nv* nodes on each list would be vaccinated, where *n* = 100,000 was the population size.

To compare the two vaccination strategies, we estimated the probability and attack rate of a major epidemic as a function of the vaccination fraction *v*. For each *v*, we generated ten independent realizations of the EPN and estimated the probability and attack rate of an epidemic by calculating the mean proportion of the population in the GIN and the GOUT, respectively. We compared strategies on two different types of contact networks: an Erdős-Rényi network with mean degree five and a scale-free network with *α* = 2 and an exponential cutoff around 50. If *p*_*k*_ denotes the probability that node has degree *k*, then *p*_*k*_ ∝ 5^*k*^/*k*! in the Erdős-Rényi network and *p*_*k*_ ∝ *k* ^−2^exp  −*k*/50 in the scale-free network.

When all nodes have the same infectiousness and susceptibility, we expect to see no difference between the two strategies because degree in the contact network is the only determinant of a node's probability of being in the GSCC. To represent the effects of variation in susceptibility and infectiousness, we allowed the transmission probability from node *i* to node *j* to be

(6) $$p_{ij}=1-e^{-100\times {\inf\;}_{i}\times {\text{sus}}_{j}},$$

where 0 < inf _*i*_ < 1 represents the infectiousness of *i* and 0 < sus_*j*_ < 1 represents the susceptibility of node *j*. We allowed inf_*i*_ and sus_*i*_ to have different beta distributions. A beta(2, 2) distribution has a single peak at 0.5, so there is little variation in infectiousness and susceptibility. A beta(.25,  .25) distribution has peaks near zero and one, so most nodes have either very high or very low infectiousness (or susceptibility). In our primary models, inf _*i*_ and sus_*i*_ were chosen independently from the specified beta distribution. To look at the effects of positive and negative correlations of infectiousness and susceptibility, we also compared the strategies in positively correlated models, where inf _*i*_ = sus_*i*_, and negatively correlated models, where inf _*i*_ = 1 − sus_*i*_.

**Results**

**Figure 7:**
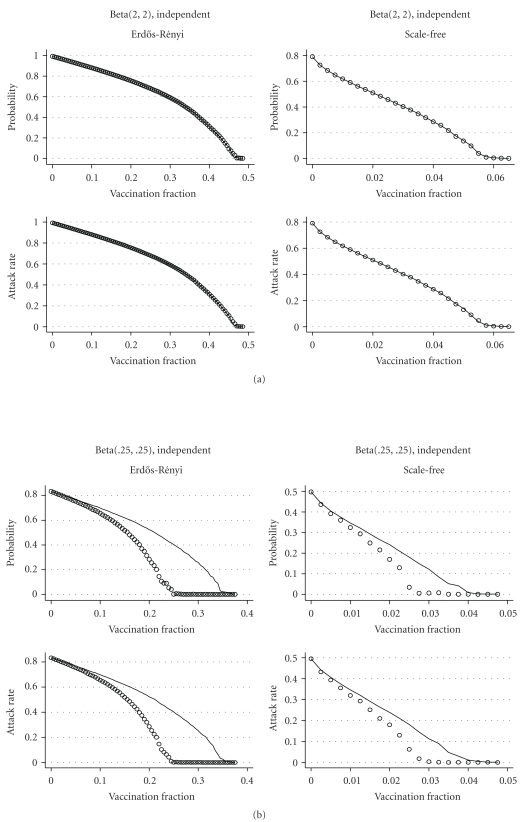
Comparison of targeting by contact network degree (lines) and targeting the GSCC (circles) in the network-based model from Section 4.2 with independent susceptibility and infectiousness. When infectiousness and susceptibility have beta(2, 2) distributions, the two strategies produce nearly identical results. When they have beta(.25,  .25) distributions, targeting the GSCC is more effective in reducing both the probability and attack rate of major epidemics on both Erdős-Rényi and scale-free networks.

**Figure 8:**
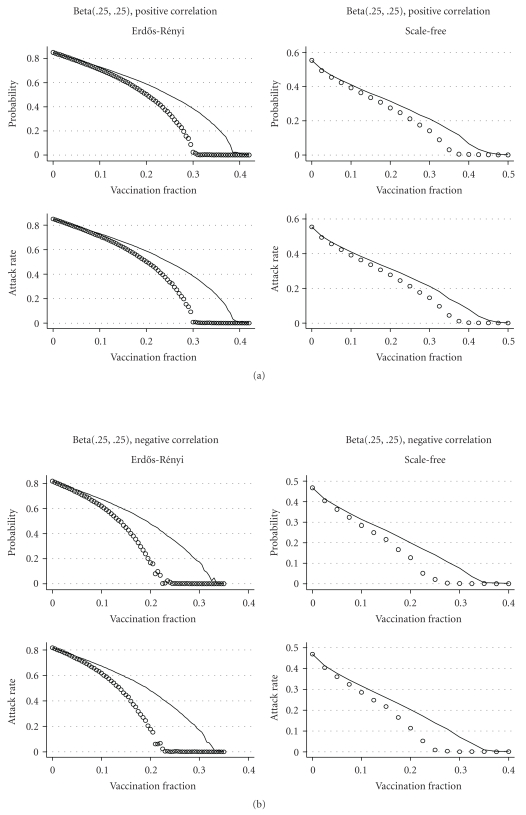
Comparison of targeting by contact network degree (lines) and targeting the GSCC (circles) in the network-based models from Section 4.2 with beta(.25,  .25) distributions of infectiousness and susceptibility and positive or negative correlations. In both cases, targeting the GSCC is more effective in reducing the probability and attack rate of an epidemic on both Erdős- Rényi and scale-free networks. In the corresponding models with beta(2, 2) distributions of infectiousness and susceptibility, targeting the GSCC and targeting by contact network degree had identical effects (not shown).

The results of the comparison for a model with independent infectiousness and susceptibility are shown in Figure 7. As expected, we see almost no difference in the effectiveness of the two strategies when inf  and sus have beta(2, 2) distributions. When inf _*i*_ and sus_*i*_ have beta(.25,  .25) distributions, targeting vaccination to the GSCC is much more effective in reducing both the probability and attack rate of a major epidemic than targeting nodes with high degree in the contact network.  Figure 8 shows similar results for models with positive and negative correlations between infectiousness and susceptibility. Targeting vaccination to the GSCC of the EPN takes advantage of information about the epidemiology of the disease that targeting to nodes with high degree in the contact network does not, and this makes a substantial difference in the effectiveness of the vaccination strategy.

## 5. Discussion

 EPNs provide a very useful intuitive point of view when thinking about the behavior of stochastic SEIR epidemic models. The “bow-tie” diagram in Figure 1 provides a simple visual explanation for the following basic facts.

- The ultimate outcome of an epidemic does not depend on where it starts.
- The probability and attack rate of an epidemic must be both zero or both positive, so there is a single epidemic threshold.
- In general, vaccinating the highly infectious (i.e., those likely to be in the GIN) will reduce the probability of a major epidemic, and vaccinating the highly susceptible (i.e., those likely to be in the GOUT) will reduce its attack rate. Vaccinating those likely to be in the GSCC will reduce both.

The ideal vaccination targets are not necessarily the most infectious or the most susceptible individuals. Instead, they are those individuals with the right combination of infectiousness and susceptibility to be effective receivers and transmitters of infection. It is precisely these nodes that hold together the GSCC of the EPN. In Section 4, we showed that targeting vaccine to nodes likely to be in the GSCC and highly connected within the GSCC was an efficient intervention strategy for both mass-action and network-based models. The correspondence between the epidemic threshold in an SEIR model and the emergence of the GSCC in its EPN makes elimination of the GSCC a necessary and sufficient condition for the elimination of disease transmission in a population.

The primary limitation of EPNs is that they are defined only for time-homogeneous SEIR models. They cannot accurately represent the final outcomes of complex, time-dependent SEIR models and interventions. For example, they cannot accurately represent seasonality, the effects of changing behavior or demographics, or the effects of an intervention that is implemented only when a certain prevalence of infection is reached. The vaccination strategies in Section 4 were all prevaccination strategies, where the population was vaccinated prior to the beginning of disease spread. If vaccination began after an epidemic had already started, as was the case in the recent influenza A(H1N1) pandemic, its effects could not be represented accurately using an EPN.

Nonetheless, EPNs generalize earlier approaches to the analysis of mass-action and network-based models, providing a simple unified framework for the analysis and implementation of time-homogeneous S(E)IR models. They are powerful theoretical and practical tools, and they represent an important application of networks in infectious disease epidemiology.

## Supplementary Material

The supplementary material contains Python code used for Section 3. It requires the NumPy and SciPy packages (http://www.scipy.org/) and the networkX package (http://networkx.lanl.gov/). The first section of the code (lines 12-333) was used for sections 3.1 (mass-action models) and 3.2 (network-based models). The epidemic probability and attack rate can be calculated using “epiP” and “epiAR” methods, respectively. The second section of the code (lines 336-848) is a largely self-contained package for creating EPNs from given networks. It allows the user to assign the rules for transmission or to use any of a number of commonly used models, including fixed generation time and exponentially distributed infectious period. The functions allow the user to create a realization of an EPN from the given network and infection process. The user can then calculate the probability or attack rate as well as the epidemic curve.

## Figures and Tables

**Table 1:** Summary of the mass-action model from Section 4.1. Subpopulation A has high infectiousness but low susceptibility, subpopulation B has average infectiousness and susceptibility (by both arithmetic and geometric mean), and subpopulation C has low infectiousness but high susceptibility. Nodes in subpopulation B have the highest probability of being in the GSCC and the highest expected number of edges connecting them to other nodes in the GSCC.

Subpopulation	A	B	C
Mean outdegree (infectiousness)	5	2.5	1.25
Mean indegree (susceptibility)	1.25	2.5	5

Pr (causes epidemic)	.951	.779	.430
Pr (infected in epidemic)	.430	.779	.951

Pr (in GSCC)	.409	.607	.409
Mean degree within GSCC	.835	.942	.835
