# Supplementary material for: Epidemic Percolation Networks, Epidemic Outcomes, and Interventions
Source: Interdiscip Perspect Infect Dis. 2011 Feb 21;2011:543520. doi: 10.1155/2011/543520 (PMC3062991; doi:10.1155/2011/543520)
Supplement: Supplementary file 1 — The supplementary material contains Python code used for Section 3. It requires the NumPy and SciPy packages (http://www.scipy.org/) and the networkX package (http://networkx.lanl.gov/). The first section of the code (lines 12-333) was used for sections 3.1 (mass-action models) and 3.2 (network-based models). The epidemic probability and attack rate can be calculated using “epiP” and “epiAR” methods, respectively. The second section of the code (lines 336-848) is a largely self-contained package for creating EPNs from given networks. It allows the user to assign the rules for transmission or to use any of a number of commonly used models, including fixed generation time and exponentially distributed infectious period. The functions allow the user to create a realization of an EPN from the given network and infection process. The user can then calculate the probability or attack rate as well as the epidemic curve. [file 543520.f1.pdf]

```
## Python code for models used in Section 3 ##
# Code for Sections 3.1 and 3.2 is on lines 12 through 333.
# Code for Section 3.4 begins on line 336.

# Epidemic percolation network generation and probability generating function
# calculations of the probability and attack rate of a major epidemic are
# based on the following references from the article:
# [8]  E. Kenah and J. M. Robins (2007). Physical Review E 76: 036113.
# [9]  E. Kenah and J. M. Robins (2007). J Theoretical Biology 249: 706-722
# [10] J. Miller (2007). Physical Review E 76: 010101(R).

## Python code for models used in Section 3.1 and 3.2. ##
# written by Eben Kenah

import math
import random
import heapq as hp

import numpy as np
import scipy.stats as stat
import scipy.special as spec
import scipy.integrate as intg
import scipy.misc as misc
import scipy.optimize as opt
import networkx as nx

# utility functions
def fixed(g, x0, tol=.000001):
    oldx = x0
    x = g(x0)
    while abs(x - oldx) > tol:
        oldx = x
        x = g(x)
    x = g(x)
    return x

def funcE(func, dist):
    # assumes dist has nonnegative support
    try:
      # standard continuous distribution
      return intg.quad(lambda x: dist.pdf(x) * func(x), 0, intg.inf)[0]
    except AttributeError:
      # user-defined discrete distribution (allows noninteger values)
      if hasattr(dist, 'pk'):
          npfunc = np.vectorize(func)
          return np.sum(dist.pk * npfunc(dist.xk))
      # standard discrete distribution
      else:
          return intg.quad(lambda x: (dist.pmf(math.floor(x))
                                     * func(math.floor(x))),
                       0, intg.inf)[0]


class epiModel():
    """
    Base class for stochastic SEIR epidemic models.  Assumes close-contact
    group in which everyone can contact everyone else with contact interval
```

```
distribution independent of population size.

"""
def __init__(self, n, CIdist, IPdist=stat.expon(),
         LPdist=stat.randint(0, 1)):
  """
  Initializes close-contact group stochastic SEIR model.

  Arguments:
      n -- number of individuals
      LPdist -- scipy.stats distribution for latent period
      IPdist -- scipy.stats distribution for infectious period
      CIdist -- scipy.stats distribution for contact interval

  """
    self.n = n
  self.popList = range(n)
    self.LPdist = LPdist
    self.IPdist = IPdist
    self.CIdist = CIdist

def infectious_contacts(self, t, i, latpd, infpd):
  """
  Infectious contact function for close-contact group model

  Arguments:
      t -- infection time of primary case
      i -- index of primary case
      latpd -- latent period of primary case
      infpd -- infectious period of primary case

  Returns:
      tuple containing (infectious contact time, index) for each
      person with whom the index case makes infectious contact

  """
  n = self.n
    iNeighbors = np.array(range(i) + range(i+1, n))
    CIlist = self.CIdist.rvs(size = n - 1)
    CItest = np.less_equal(CIlist, infpd)
    return zip(t + latpd + CIlist[CItest], iNeighbors[CItest])

def epidemic(self, importedInfections=None):
  """
  Runs an epidemic to completion.

  Arguments:
      importedInfections -- list containing (index, importation time)
                      for each possible imported infection

  """
    # initialize data lists
    exptimes = dict([(i, -1) for i in range(self.n)])
    LPlist = list(self.LPdist.rvs(size = self.n))
    IPlist = list(self.IPdist.rvs(size = self.n))
  ninf = 0
    epiHeap = []
```

```
    hp.heapify(epiHeap)
  if not importedInfections:
      importedInfections = [(random.choice(self.popList), 0)]

    # run epidemic
  infectious_contacts = self.infectious_contacts
    for (i, t) in importedInfections:
        hp.heappush(epiHeap, (t, i, self.n))
    while epiHeap:
        t, i, vi = hp.heappop(epiHeap)
        if exptimes[i] == -1:
            # record data for i
            exptimes[i] = t
            latpd = LPlist.pop()
            infpd = IPlist.pop()
        ninf += 1

            # generate infectious contacts from i to neighbors
            contacts = infectious_contacts(t, i, latpd, infpd)
            for (tij, j) in contacts:
                if exptimes[j] == -1 or exptimes[j] > tij:
                    hp.heappush(epiHeap, (tij, j, i))
  self.ninf = ninf

def stopped_epidemic(self, importedInfections=None, stopSize=1000):
  """
  Runs an epidemic, stopping with extinction or 'stopSize' infections.

  Arguments:
      importedInfections -- list containing (index, importation time)
                      for each possible imported infection
      stopSize -- the number of infections after which the epidemic
              will halt
      itrLimit -- the maximum number of attempts that will be made to
              obtain at least 'stopSize' infections

  """
    # initialize data lists
    exptimes = dict([(i, -1) for i in xrange(self.n)])
    LPlist = list(self.LPdist.rvs(size = self.n))
    IPlist = list(self.IPdist.rvs(size = self.n))
  ninf = 0
    epiHeap = []
    hp.heapify(epiHeap)
  if not importedInfections:
      importedInfections = [(random.choice(self.popList), 0)]

    # run epidemic
    for (i, t) in importedInfections:
        hp.heappush(epiHeap, (t, i, self.n))
    while epiHeap and ninf < stopSize:
        t, i, vi = hp.heappop(epiHeap)
        if exptimes[i] == -1:
            # record data for i
            exptimes[i] = t
            latpd = LPlist.pop()
            infpd = IPlist.pop()
```

```
            ninf += 1

                # generate infectious contacts from i to neighbors
                contacts = self.infectious_contacts(t, i, latpd, infpd)
                for (tij, j) in contacts:
                    if exptimes[j] == -1 or exptimes[j] > tij:
                        hp.heappush(epiHeap, (tij, j, i))
      self.ninf = ninf

# mass-action models
class exponCI_massAction_epiModel(epiModel):
    def __init__(self, n, CIbeta, *args, **kwargs):
      CIdist = stat.expon(scale = 1./CIbeta)
      epiModel.__init__(self, n, CIdist, *args, **kwargs)
        self.CIbeta = CIbeta
        self.R0 = CIbeta * self.IPdist.stats('m')

    def infectious_contacts(self, t, i, latpd, infpd):
        cumhazard = self.CIbeta * infpd
        contactnum = stat.poisson(cumhazard).rvs()
        contacts = random.sample(range(i) + range(i + 1, self.n), contactnum)
        CIlist = stat.uniform(scale = infpd).rvs(size = contactnum)
        return zip(t + latpd + CIlist, contacts)

    def epiP(self):
      IPdist, CIbeta = self.IPdist, self.CIbeta
      exp = math.exp
      def G1out(y):
           def g1out(infpd):
            infpdCH = CIbeta * infpd
            return exp(infpdCH * (y - 1))
           return funcE(g1out, IPdist)
      v = fixed(G1out, .0001)
      # G0out = G1out because of no variation in susceptibility
      return 1 - v

    def epiAR(self):
      IPdist, CIbeta = self.IPdist, self.CIbeta
      exp = math.exp
      meanCH = CIbeta * IPdist.stats('m')
      def G1in(x):
          return exp(meanCH * (x - 1))
      v = fixed(G1in, .0001)
      # G0in = G1in because of no variation in susceptibility
      return 1 - v

class WeibullCI_massAction_epiModel(epiModel):
    def __init__(self, n, CIalpha, CIbeta, *args, **kwargs):
        CIdist = stat.weibull_min(CIalpha, scale=1./CIbeta)
      epiModel.__init__(self, n, CIdist, *args, **kwargs)
        self.CIalpha = CIalpha
        self.CIbeta = CIbeta
      self.R0 = intg.quad(lambda x: (self.IPdist.pdf(x)
                              * (CIbeta * x)**CIalpha),
                      0, intg.inf)[0]

    def infectious_contacts(self, t, i, latpd, infpd):
```

```
        cumhazard = (self.CIbeta * infpd)**self.CIalpha
        contactnum = stat.poisson(cumhazard).rvs()
        contacts = random.sample(range(i) + range(i + 1, self.n), contactnum)
        CIlist = (1./self.CIbeta
              * stat.uniform(scale = cumhazard).rvs(size = contactnum)
              **(1./self.CIalpha))
        return zip(t + latpd + CIlist, contacts)

    def epiP(self):
      IPdist, CIalpha, CIbeta = self.IPdist, self.CIalpha, self.CIbeta
      exp = math.exp
      def G1out(y):
           def g1out(infpd):
            infpdCH = (CIbeta * infpd)**CIalpha
            return exp(infpdCH * (y - 1))
           return funcE(g1out, IPdist)
      v = fixed(G1out, .0001)
      # G0out = G1out because of no variation in susceptibility
      return 1 - v

    def epiAR(self):
      IPdist, CIalpha, CIbeta = self.IPdist, self.CIalpha, self.CIbeta
      exp = math.exp
      meanCH = funcE(lambda infpd: (CIbeta * infpd)**CIalpha, IPdist)
      def G1in(x):
          return exp(meanCH * (x - 1))
      v = fixed(G1in, .0001)
      # G0in = G1in because of no variation in susceptibility
      return 1 - v

# network-based models base class and subclasses
class network_epiModel(epiModel):
    def __init__(self, network, CIdist, *args, **kwargs):
        """
        network  -- NetworkX network for the spread of infection
        CIdist -- a ``frozen'' Scipy.stats distribution for contact interval

        """
      n = network.order()
      epiModel.__init__(self, n, CIdist, *args, **kwargs)
      self.network = network
      self.popList = network.nodes()
        self.meanD = 2 * self.network.size() / float(self.network.order())
      self.Dseq = np.array(network.degree())
      self.tildeD = np.mean(self.Dseq * (self.Dseq - 1) / self.meanD)
      self.T = funcE(self.CIdist.cdf, self.IPdist)
      self.R0 = self.T * self.tildeD

    def infectious_contacts(self, t, i, latpd, infpd):
        iNeighbors = np.array(self.network.neighbors(i))
        CIlist = self.CIdist.rvs(size = self.network.degree(i))
        CItest = np.less_equal(CIlist, infpd)
        return zip(t + latpd + CIlist[CItest], iNeighbors[CItest])

    def epiP(self):
      IPdist, CIdist, meanD = self.IPdist, self.CIdist, self.meanD
      Dhist = np.bincount(self.Dseq)/float(self.n)
```

```
      Dlist = np.arange(len(Dhist))
      def CN_pgf1(z):
          return np.sum(Dlist * Dhist * z**(Dlist - 1))/meanD
      def G1out(y):
           def g1out(infpd):
            infpdT = CIdist.cdf(infpd)
            return CN_pgf1(1 - infpdT + infpdT * y)
           return funcE(g1out, IPdist)
      v = fixed(G1out, .0001)
      def CN_pgf0(z):
          return np.sum(Dhist * z**Dlist)
      def g0out(infpd):
          infpdT = CIdist.cdf(infpd)
          return CN_pgf0(1 - infpdT + infpdT * v)
      epiQ = funcE(g0out, IPdist)
      return 1 - epiQ

    def epiAR(self):
      IPdist, CIdist, meanD = self.IPdist, self.CIdist, self.meanD
      T = self.T
      Dhist = np.bincount(self.Dseq)/float(self.n)
      Dlist = np.arange(len(Dhist))
      def CN_pgf1(z):
          return np.sum(Dlist * Dhist * z**(Dlist - 1))/meanD
      def G1in(x):
          def g1in(infpd):
            return CN_pgf1(1 - T + T * x)
          return funcE(g1in, IPdist)
      v = fixed(G1in, .0001)
      def CN_pgf0(z):
          return np.sum(Dhist * z**Dlist)
      def g0in(infpd):
          return CN_pgf0(1 - T + T * v)
      epiS = funcE(g0in, IPdist)
      return 1 - epiS

class exponCI_network_epiModel(network_epiModel):
    def __init__(self, network, CIbeta, *args, **kwargs):
      self.CIbeta = CIbeta
      CIdist = stat.expon(scale = 1./CIbeta)
      network_epiModel.__init__(self, network, CIdist, *args, **kwargs)

class WeibullCI_network_epiModel(network_epiModel):
    def __init__(self, network, CIalpha, CIbeta, *args, **kwargs):
      self.CIalpha = CIalpha
      self.CIbeta = CIbeta
      CIdist = stat.weibull_min(CIalpha, scale = 1./CIbeta)
      network_epiModel.__init__(self, network, CIdist, *args, **kwargs)


## Python code for models used in Section 3.4 ##
# written by Joel C. Miller

import networkx
import random
import math
```

```
__author__ = """Joel C. Miller joel.c.miller@gmail.com"""

"""
Most of this code was written by Joel C. Miller.  A few pieces were
created in collaboration with Eben Kenah.  The purpose of this code is
to use the Epidemic Percolation Network structure [Kenah & Robins:
Network-based analysis of stochastic SIR epidemic models with random
and proportionate mixing, J Theor Biol; J C Miller: The spread of
infectious diseases through clustered populations, Royal Society
Interface] in order to efficiently analyze the structure of SIR
epidemics in static networks.

We give a quick explanation of an Epidemic Percolation Network:

Given a static network, we have several options for how to simulate an
epidemic.  The most obvious is to begin with an infected node,
consider each neighbor and generate a random number based on
properties of the contact.  If the random number is small enough, we
infect that neighbor.  The process repeats.  For many purposes this
process is very inefficient.

In the approach above we roll a die for each contact once the disease
has reached one of them.  However, we could just as easily roll that
die for each contact before the epidemic simulation begins.  We ask
the question: assuming $u$ gets infected, does s/he infect $v$?  If
yes, then we place a directed edge from $u$ to $v$ (we can even assign
a weight to represent how long the infection takes from the time $u$
becomes infected).  After we have done this for every contact (in both
directions), we have created the Epidemic Percolation Network.  We now
choose the index case.  The disease spreads from the index case along
the pre-calculated edges (with appropriate time spent for each
transmission).

The Epidemic Percolation Network (EPN) gives us a static structure we
can study.  In general, aside from pathological cases, if
transmissibility is high enough, the EPN has a single unique giant
strongly-connected-component (scc).  The set of nodes from which the
scc can be reached is called $G_{in}$ (and includes the scc).  The set
of nodes reachable from the scc is $G_{out}$ (and also includes the
scc).  The proportion of nodes in $G_{in}$ is a close approximation
[error roughly (log N)/N] of the probability of an epidemic and the
proportion in $G_{out}$ is a close approximation of the attack rate.

here is an example of using this code for a simple outbreak on a
erdos reyni network where the transmissibility T=0.8 is fixed.

from networkx import *
from epidemic_code import *
N=100000  #set network size
G=fast_gnp_random_graph(N, 4./(N-1.0))   #create network expected degree 4
EPN = create_EPN_fixed_transmissiblity(G,0.8)  #create (directed) EPN with T=0.8
[P,A] = get_prob_and_size(EPN)                 #find P and A.  Since T
                                               #is constant, they will
                                               #be almost identical.
```

```
[infection_curve,times]= create_epidemic_curve(EPN,23)  #find the epidemic curve
for an epidemic starting at node 23.  Since no recovery times are specified, it
assumes recovery happens at time after one unit of time.  The default EPN
created has weight 1 for each edge, so it also assumes that infection happens
after one unit of time.

changes:
v0.1 -> v0.2
corrected bug referencing edge[2] in output_EPN and output_dendrogram

corrected but in fixed_rec_exp_inf_infection which did not give correct
infection duration and also another that had a time2infect1, rather than
time2infect.

modified EPN creation routine to allow directed networks as input.

removed I and S (and similar) and replaced them with node attributes:
node attributes:
  infection_duration
  type
edge attributes:
  time_to_infection
  type

to do this, eliminated PIS which created dicts mapping node to I and S
and replaced with type_assignment which create a dict for each node giving I and
S or other appropriate vars


have changed 'parameters' from a list to a dict.

"""



######   EPN CREATION CODE
#We start with code for creating EPNs

def create_EPN(G,type_assignment,attempt_infection,parameters):

    """
Creates an EPN from the graph or DiGraph G, using various rules we
     might want to apply to the infection process.  It returns just the EPN.

G: the underlying network on which the epidemic spreads

type_assignment: A generic function which takes EPN, a node name, and any
     parameters and then adds the node to the EPN with appropriate attributes:
     e.g., duration of infection, infectiousness, susceptibility, type, etc.

attempt_infection: A function of the form
     attempt_infection(u,v,I,S,parameters) where I and S are
     dictionaries giving I[u] and S[v], the infectiousness and
     susceptibility of u and v respectively.  It then determines
     whether u will infect v, returning [True,time] if so and [False]
     otherwise (the only important part of the second result is that
     the first entry evaluates to False).  Here time is the time it
     takes for infection to happen.  If this is unimportant, it can be
```

```
     set to 1.

parameters: Any parameters that type_assignment and attempt_infection might
       need.  This is a dict

return_weights: optional argument.  If True then returns [EPN,I,S].  If False or
       unspecified, just returns EPN

A number of routines have been developed that use this to create EPNs:

create_EPN_fixed_transmissibility(G,T)
       create an EPN with constant transmissibility on the graph G.

create_EPN_exponential_rec_and_inf(G,gamma,beta)
       create an EPN with constant recovery rate gamma and constant
       infection rate beta.

create_EPN_fixed_recovery_exponential_inf(G,tau,beta)
       create an EPN where everyone recovers after tau units of time
       and infectiousness is constant at rate beta.
    """

    if type(G).__name__ not in ['Graph' , 'DiGraph', 'MultiGraph']:
        #not sure if algorithm works if other graph type used.
        raise networkx.NetworkXError("Bad type %s for input
network"%type(G).__name__)
#    if type(G).__name__ == 'MultiGraph':
#        print 'warning, received %s, proceeding as normal'%type(G).__name__
    EPN=networkx.DiGraph(weighted=True) #will give error if using
                                    #networkx prior to 0.99
    node_assignment(G,EPN,type_assignment,parameters)
    edge_assignment(G,EPN,parameters,attempt_infection) #need to send G so that
we can grab any edge attributes.
    return EPN



def create_EPN_weighted_edges(G,type_assignment,attempt_infection,parameters):
    """
    The new structure of create_EPN allows weighted edges.  So I'm just keeping
this code for compatibility reasons.
    """
    print "create_EPN_weighted_edges is obsolete - use create_EPN"
    EPN = create_EPN(G,type_assignment,attempt_infection,parameters)
    return EPN

def create_EPN_preassigned_weights(G,I,S,attempt_infection,parameters):
    print "create_EPN_preassigned_weights is obsolete - use create_EPN"
    EPN = create_EPN(G,type_assignment,attempt_infection,parameters)
    return EPN

def node_assignment(G,EPN,type_assignment,parameters):
    nodes = G.nodes_iter()
    for node in nodes:
        type_assignment(EPN,node,G.node[node],parameters)

def edge_assignment(G,EPN,parameters,attempt_infection):
```

```
    edges = G.edges_iter()
    if type(G).__name__ == 'DiGraph':
        for edge in edges:

attempt_infection(EPN,edge[0],edge[1],G.get_edge_data(edge[0],edge[1]),parameter
s)
    elif type(G).__name__ in ['Graph', 'MultiGraph']:
        for edge in edges:

attempt_infection(EPN,edge[0],edge[1],G.get_edge_data(edge[0],edge[1]),parameter
s)

attempt_infection(EPN,edge[1],edge[0],G.get_edge_data(edge[1],edge[0]),parameter
s)
    else:
        raise networkx.NetworkXError("Bad type %s for input
network"%type(G).__name__)



'''
basic structure of an attempt_infection code:
calculate time to infection, or any other variable needed to determine if
infection occurs.
Find if infection occurs.
If so, add edge to EPN with appropriate data attached (e.g., time_to_infection)
'''



#### Done with basic EPN creation code.  Now dealing with specific cases.

#constant infection and recovery rates - consistent with ODE models

def exp_rec_and_inf_type_assignment(EPN,node,node_data,parameters):
    gamma = parameters['gamma']
    tau = random.expovariate(gamma)
    EPN.add_node(node,infection_duration=tau)
    EPN.node[node].update(node_data)

def exp_rec_and_inf_attempt_inf(EPN,node0,node1,edge_data,parameters):
    beta = parameters['beta']
    time2infect = random.expovariate(beta)
    if time2infect<EPN.node[node0]['infection_duration']:
        EPN.add_edge(node0,node1,time_to_infection=time2infect)
        EPN[node0][node1].update(edge_data)

def create_EPN_exponential_rec_and_inf(G,gamma,beta):
    """
    creates an EPN on network G corresponding to constant recovery
    rate (gamma) and constant infectiousness (beta).  The time to
    recovery is exponentially distributed.  The time to infection is
    also exponentially distributed, but all infections happening after
    recovery are discarded.
    """
    #return_value is either just EPN or [EPN,I,S]
    parameters = {'gamma':gamma, 'beta':beta}
```

```
    return
create_EPN(G,exp_rec_and_inf_type_assignment,exp_rec_and_inf_attempt_inf,paramet
ers)


#epidemics with fixed transmissibility
def fixed_trans_type_assignment(EPN,node,node_data,parameters):
    EPN.add_node(node,infection_duration=1)
    EPN.node[node].update(node_data)
def fixed_trans_infection(EPN, node0, node1,edge_data,parameters):
    T=parameters['transmissibility']
    if random.random()<T:
        EPN.add_edge(node0,node1,time_to_infection=1)
        EPN[node0][node1].update(edge_data)
def create_EPN_fixed_transmissibility(G,T):
    """
    creates an EPN for the network G with transmissibility T.  Everything is
divided into generations.
    """
    #return_value is either just EPN or it is [EPN, I, S]
    parameters = {'transmissibility':T}
    return
create_EPN(G,fixed_trans_type_assignment,fixed_trans_infection,parameters)



#epidemics with fixed recovery time and constant infection rate --- this is a
special case of fixed transmissibility, but allows us to assign an infection
time

def fixed_rec_exp_inf_type_assignment(EPN,node,node_data,parameters):
    duration = parameters['infection_duration']
    EPN.add_node(node,infection_duration=duration)
    EPN.node[node].update(node_data)
def fixed_rec_exp_inf_infection(EPN, node0, node1,edge_data,parameters):
    duration = parameters['infection_duration']
    beta = parameters['beta']
    time2infect = random.expovariate(beta)
    if time2infect<duration:
        EPN.add_edge(node0,node1,time_to_infection=time2infect)
        EPN[node0][node1].update(edge_data)
def create_EPN_fixed_recovery_exponential_inf(G,tau,beta):
    """
    create an EPN from the graph G assuming a fixed infection period
    tau and constant infectiousness beta
    """
   #return_value is either just EPN or it is [EPN, I, S]
    parameters = {'infection_duration':tau,'beta':beta}
    return
create_EPN(G,fixed_rec_exp_inf_type_assignment,fixed_rec_exp_inf_infection,param
eters)
```

```
#shown by trapman to give lower bound for probability in case where
susceptibility is homogeneous.  I suspect it's also the lower bound if
susceptibility allowed to vary.

def extreme_het_type_assignment(EPN,node,node_data,parameters):

EPN.add_node(node,rel_infectiousness=random.random(),rel_susceptibility=random.r
andom())
    EPN.node[node].update(node_data)
def extreme_het_inf_infection(EPN, node0, node1,edge_data,parameters):
    T = parameters['transmissibility']
    if EPN.node[node0]['rel_infectiousness']<T:
        EPN.add_edge(node0,node1,time_to_infection=1)
        EPN[node0][node1].update(edge_data)
def extreme_het_sus_infection(EPN,node0,node1,edge_data,parameters):
    T = parameters['transmissibility']
    if EPN.node[node1]['rel_susceptibility']<T:
        EPN.add_edge(node0,node1,time_to_infection=1)
        EPN[node0][node1].update(edge_data)

def create_EPN_extreme_het_inf(G,T):
    parameters={'transmissibility':T}
    return create_EPN(G, extreme_het_type_assignment, extreme_het_inf_infection,
parameters)

def create_EPN_extreme_het_sus(G,T):
    parameters={'transmissibility':T}
    return create_EPN(G, extreme_het_type_assignment, extreme_het_sus_infection,
parameters)




def get_prob_and_size(EPN):
    """
    Calculates the probability and attack rate of epidemics by finding
    the relative size of the in-component of the largest strongly
    connected component (this is the probability of an epidemic P) and
    finding the relative size of the out-component of the largest
    strongly connected component (this is the attack rate A).

    Note that these in- and out-components include the strongly
    connected component.

    Warning - this returns the sizes for the largest
    strongly-connected-component, whether or not it is a giant
    component.

    Returns [P,A]
    """
    N=EPN.order()
    scc_list = networkx.strongly_connected_components(EPN)
    start_node = scc_list[0][0]
```

```
    out_component = networkx.dfs_preorder(EPN,start_node)
    in_component = networkx.dfs_preorder(EPN,start_node,reverse_graph = True)

    A = len(out_component)*1.0/N
    P = len(in_component)*1.0/N

    return [P,A]
```

```
def create_epidemic_curve(EPN, source=None, cum_inc = False, stratify = False):
#this is effectively Dijstra's algorithm, with additional info on the recovery
times.  It returns a dictionary with the times that recovery/infection occurs,
and the current number infected at that time.  If source is None, it finds a
random source from which the largest scc in EPN can be reached.  If cum_inc is
True, then rather than just giving number infected, it also returns cumulative
incidence.  If you want to know number still susceptible, just subtract
cumulative infections from population.
    events = {}
    if source == None:
        scc_list = networkx.strongly_connected_components(EPN)
        start_node = scc_list[0][0]
        in_component = networkx.dfs_preorder(EPN,start_node,reverse_graph =
True)
        source = random.choice(in_component)

    """WARNING WARNING: need to edit single_source_dijkstra to accept
weight='weight' as an optional argument.  Then change all occurrences of
'weight' to weight"""

    [distances,paths] = networkx.single_source_dijkstra(EPN, source, weight =
'time_to_infection')
    for node in distances.keys():  #key is node, distance[key] is distance
following node.
        events[distances[node]] = events.get(distances[node],0)+1

    if cum_inc:  #if also returning cumulative incidence, not just infection
curve.
        infections = {}
        for node in distances.keys():
            infections[distances[node]] = infections.get(distances[node],0)+1
        cum_times = infections.keys()
        cum_times.sort()
        cumulative_curve = [0]
        for time in cum_times:
            newvalue = cumulative_curve[-1]+infections[time]
            cumulative_curve.append(newvalue)
        cumulative_curve.pop(0)

    for node in distances.keys():
```

```
        events[distances[node]+EPN.node[node].get('infection_duration',1)] =
events.get(distances[node]+EPN.node[node].get('infection_duration',1),0)-1

    tmp = events.items()
    tmp.sort()

    infection_curve = []
    times=[]
    current_count = 0
    for event in tmp:
        current_count += event[1]
        infection_curve.append(current_count)
        times.append(event[0])
    if cum_inc:
        return [infection_curve,times,cumulative_curve,cum_times]
    else:
        return [infection_curve,times]


def create_epidemic_curve_stratified(EPN, source=None, cum_inc = False): #this
is effectively Dijstra's algorithm, with additional info on the recovery times.
It returns a dictionary with the times that recovery/infection occurs, and the
current number infected at that time.  If source is None, it finds a random
source from which the largest scc in EPN can be reached.  If cum_inc is True,
then rather than just giving number infected, it also returns cumulative
incidence.  If stratify is true, it also returns stratifications by 'type'.  If
you want to know number still susceptible, just subtract cumulative infections
from population.
    events = {}
    if source == None:
        scc_list = networkx.strongly_connected_components(EPN)
        start_node = scc_list[0][0]
        in_component = networkx.dfs_preorder(EPN,start_node,reverse_graph =
True)
        source = random.choice(in_component)


    """WARNING WARNING: need to edit single_source_dijkstra to accept
weight='weight' as an optional argument.  Then change all occurrences of
'weight' to weight"""

    [distances,paths] = networkx.single_source_dijkstra(EPN, source, weight =
'time_to_infection')
    for node in distances.keys():  #key is node, distance[key] is distance
following node.
        type = EPN.node[node]['type']
        if not events.has_key(type):
            events[type]={}
        events[type][distances[node]] = events[type].get(distances[node],0)+1

    if cum_inc:  #if also returning cumulative incidence, not just infection
curve.
        infections = {}
        for node in distances.keys():
            type = EPN.node[node]['type']
            if not infections.has_key(type):
```

```
                infections[type][distances[node]] =
infections[type].get(distances[node],0)+1
        cum_times={}
        cumulative_curve={}
        for type in infections.keys():
            cum_times[type] = infections.keys()
            cum_times[type].sort()
            cumulative_curve[type] = [0]
            for time in cum_times[type]:
                newvalue = cumulative_curve[-1]+infections[time]
                cumulative_curve[type].append(newvalue)
            cumulative_curve[type].pop(0)

    for node in distances.keys():
        type = G[node]['type']
        events[type][distances[node]+EPN.node[node].get('infection_duration',1)]
=
events[type].get(distances[node]+EPN.node[node].get('infection_duration',1),0)-1

    tmp = {}
    for type in events.keys():
        tmp[type] = events[type].items()
        tmp[type].sort()

    infection_curve = {}
    times={}
    for type in tmp.keys():
        current_count = 0
        infection_curve[type]=[]
        times[type]=[]
        for event in tmp[type]:
            current_count += event[1]
            infection_curve[type].append(current_count)
            times[type].append(event[0])
    if cum_inc:
        return [infection_curve,times,cumulative_curve,cum_times]
    else:
        return [infection_curve,times]
```

```
#####   MAKE PREDICTIONS - ANALYTIC #####

#predictions assume configuration model type networks.
#
# Method basically follows J C Miller: Epidemic size and probability
# in populations with heterogeneous infectiousness and susceptibility.
# PRE 76 010101(R) 2007

def get_Pk(G):
    P = {}
    order = G.order()
    inv_order = 1./order
    for node in G.nodes_iter():
        k = G.degree(node)
        P[k] = P.get(k,0) + inv_order
    return P


def simple_fixed_trans_prob_size_prediction(G,T):
    Pk = get_Pk(G)
    [P,A]= fixed_trans_pgf_probsize_prediction(T,Pk)
    return [P,A]
def fixed_trans_pgf_probsize_prediction(T,Pk,iterations=1000):
    x=0
    PTo={}
    PTo[T]=1
    for counter in range(iterations):
        x=h(PTo,Pk,x)
    P = 1- f(PTo,Pk,x)
    return [P,P]


def theta(T,x):
    return 1 - T + T*x

def f(PT,Pk,x):
    fx = 0
    for T in PT.keys():
        tmp = 0
        for k in Pk.keys():
#            tmp = tmp + (1+T*(x-1))**k*Pk[k]
            tmp = tmp + theta(T,x)**k*Pk[k]
        fx += PT[T]*tmp
    return fx

def h(PT,Pk,x):
    avek=0
    for k in Pk.keys():
        avek+= k*Pk[k]
    hx = 0
    for T in PT.keys():
        tmp = 0
        for k in Pk.keys():
            if k>0:
#                tmp = tmp + (1+T*(x-1))**(k-1)*k*Pk[k]
                tmp = tmp + theta(T,x)**(k-1)*k*Pk[k]
```

```
    hx += PT[T]*tmp/avek
return hx
```
